# Influence of Crystal Modifier Content on Ni-Cu Catalysts Dedicated to the Hydrogen Evolution Reaction

**DOI:** 10.3390/ma18112499

**Published:** 2025-05-26

**Authors:** Katarzyna Skibińska, Anna Kula, Dawid Kutyła, Marek Wojnicki, Piotr Żabiński

**Affiliations:** Faculty of Non-Ferrous Metals, AGH University of Krakow, al. Adama Mickiewicza 30, 30-059 Krakow, Poland; kula@agh.edu.pl (A.K.); kutyla@agh.edu.pl (D.K.); marekw@agh.edu.pl (M.W.); zabinski@agh.edu.pl (P.Ż.)

**Keywords:** Ni-Cu alloys, crystal modifier, hydrogen evolution reaction

## Abstract

Ammonium chloride is a commonly used crystal modifier allowing the production of conical structures. Metals and alloys synthesized in the form of cones show enhanced catalytic activity and active surface area. Ni-Cu alloys as candidates for catalysts in the hydrogen evolution reaction were synthesized using a one-step method. The influence of the NH_4_Cl content on morphology, chemical and physical composition, wettability, roughness, and catalytic properties was analyzed using many techniques, including, inter alia, Scanning Electron Microscopy, X-ray Diffraction, Atomic Force Microscopy, and Linear Sweep Voltammetry. The proposed deposition parameters allow the successful synthesis of conical Ni-Cu structures with promising catalytic activity compared with other coatings of these alloys. The lowest determined value of the Tafel slope is 79 mV/dec for the sample deposited from the electrolyte with 40 g/L NH_4_Cl.

## 1. Introduction

Ni-Cu alloys are an interesting candidate for applications in hydrogen evolution reactions (HERs) [1,2,3,4,5,6,7,8]. The surface morphology [9] and roughness [10] are affected by the concentration of Cu^2+^ ions and the pH of the electrolyte. J. Niu et al. [11] proposed the self-etching electrodeposition of Ni-Cu alloys by quick deposition of Ni on Cu substrates from electrolytes containing 0.1 M NiCl_2_⋅6H_2_O, 0.2 M NH_4_Cl, and 0.43 M NaCl. Due to the designed concentration of ammonium chloride and sodium chloride, copper substrates corroded during the electrodeposition into cupric–ammonium complexes and then reduced to form a Cu atom and eventually co-deposited with nickel atoms on the substrates. The fabricated Ni-Cu alloys show exquisite catalytic activity and stability in hydrogen and oxygen evolution reactions. Electrodeposited alloys show similar room temperature electrical resistivity, thermopower, and Curie temperature to metallurgically processed Ni-Cu alloys [12]. They can also act as a protective corrosion barrier on mild steel [13]. The fabrication of Ni-Cu alloys by electrodeposition from ionic liquid [14,15,16,17] or by pulsed deposition [18,19,20,21] is also often used. Nickel and copper can also be electrodeposited with other metals, e.g., Mo [22,23,24], Co [25], Zn, and Cd [26], and form ternary alloys. Conical Ni-Cu structures have already been synthesized by the deposition of Cu on Ni cones and then further annealing of the obtained coating [27]. However, this process caused the rounding of cones and a decrease in catalytic activity due to the changes in the morphology and chemical composition of the surface. Ni-Cu alloys, willingly tested by researchers of HERs, are still not applied in industrial water splitting reactions. The proposed alloys should show satisfactory activity and corrosion resistance in harsh environments. A composite-coated, commercially available Raney nickel alloy powder composed of nickel and aluminum (50/50) was prepared from a modified Watts-type bath [28]. For these electrodes, he values of hydrogen overvoltages were almost constant for more than one and a half years in a 35 wt.% NaOH at 90 °C. The electrodeposited nanocrystalline Ni–Fe alloy is an efficient electrode material with good corrosion resistance in 6 M KOH [29]. The composite of Fe–Ni–graphene was also tested in 6 M KOH and showed significantly higher activity in the HER than the binary Fe–Ni alloy [30]. D. Gao [31] and others first prepared nickel mesh by electroetching in seawater and then deposited nickel nanowires onto this mesh. The prepared electrode delivered a high current density of 800 mA/cm^2^ at 2.0–2.1 V. Moreover, the current density was stable after a 100 h test at 500 mA/cm^2^. The focus on Ni and its alloys dedicated to industrial HERs is strong [32]. Therefore, new approaches to increase its catalytic properties have been tested, e.g., transition-metal phosphide catalysts [33].

The one-step method is a widely used technique that allows the fabrication of conical structures during a single electrodeposition process from electrolytes containing the addition of a crystal modifier. In this way, the active surface area of the samples and their catalytic activity can be enhanced. Numerical simulations show that the presence of a magnetic field can also support the growth of conical structures [34]. Ammonium chloride is widely used in the synthesis of conical structures, e.g., Ni [35], Co [36], and Co-Fe [37], where it acts like a crystal modifier and blocks the horizontal direction of growth. However, the literature review shows that the presence of this chemical component can also affect other properties of the material. For Zn−Ni films electrodeposited from a choline chloride-based ionic liquid [38], the addition of NH_4_Cl suppresses the incorporation of Zn into the coating. During the electrodeposition of Fe at a pH equal to 1, the addition of ammonium chloride and malonic acid stopped the formation of Fe oxides [39]. K. H. Lee and others [40] analyzed the influence of the NH_4_Cl concentration on the magnetic properties of Co(P) alloy films. They noticed that an increase in the concentration of ammonium chloride causes the fabrication of a coating with a larger grain size and therefore lower coercivities. For the deposition of Al from the DMSO_2_–AlCl_3_ electrolyte [41], the addition of NH_4_Cl allows the electrodeposition of Al at a potential where side reactions are not present.

In this work, the influence of NH_4_Cl content on the morphology, chemical and phase composition, roughness, wettability, and electrocatalytic activity of alloys was investigated. Several characterization techniques were used, such as Scanning Electron Microscopy (SEM) with Energy-dispersive Spectroscopy (EDS), X-ray Diffraction (XRD), and Atomic Force Microscopy (AFM). The proposed method of Ni-Cu cones synthesis has not been described in the literature before. The performed experiments show other limitations of the one-step method.

## 2. Materials and Methods

**Figure 1:**
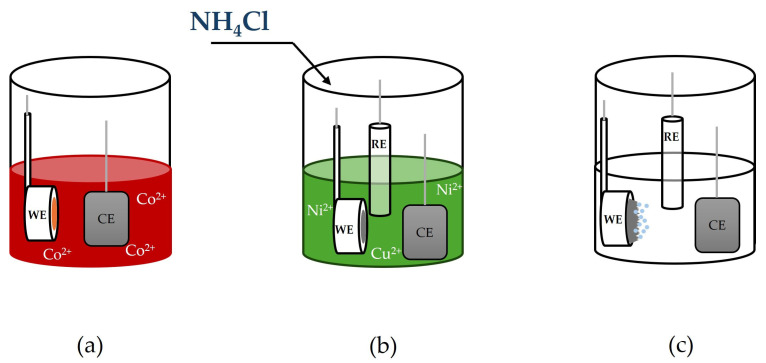
Scheme of (**a**) deposition of Co on Cu substrate, (**b**) synthesis of Ni-Cu cones, and (**c**) hydrogen evolution reaction. The actual scale is not maintained (WE—working electrode, CE—counter electrode, and RE—Reference Electrode). The scheme is for illustrative purposes only.

The scheme of Ni-Cu electrode preparation is shown in Figure 1. Ni-Cu alloys were electrodeposited on Cu substrates covered with Co (Cu/Co) (Figure 1a) to avoid any influence of the substrate’s signal on further analyses of the synthesized alloys. Cobalt layers were deposited for 15 min from the electrolyte containing 200 g/L CoCl_2_∙6H_2_O at 10 mA/cm^2^.

CuCl_2_∙2H_2_O was added to the solution for Ni cone synthesis, containing 200 g/L NiCl_2_∙6H_2_O and 100 g/L H_3_BO_3_, to obtain Ni-Cu alloys, and NH_4_Cl to deposit these alloys in the form of cones (Figure 1b). The pH of the electrolyte was equal to 4. Each sample was deposited for 5 min at 20 mA/cm^2^ at 60 °C ± 1 °C. The Cu/Co substrate was a working electrode, the saturated calomel electrode (SCE) was a reference electrode, and the Pt foil was a counter electrode. The process parameters were chosen based on the literature review [42].

The morphology of the synthesized coatings was analyzed using a Hitachi SU-70 SEM (Hitachi, Tokyo, Japan). All the specimens were tilted 40° during SEM observation for better visualization. The chemical compositions of the coatings were studied using an SEM JEOL-6000 Plus (JEOL, Tokyo, Japan) equipped with an Energy Dispersive X-ray Spectrometer (EDS). The average roughness S_a_ of the deposited alloys was characterized using Atomic Force Microscopy (AFM) NTegra Aura NT MDT (Moscow, Russia) in a semicontact mode using an NSG03 tip (TipsNano, Tallinn, Estonia). X-ray Diffraction (XRD) scans were performed with a Rigaku MiniFlex II apparatus (Tokyo, Japan) equipped with a Cu lamp with a wavelength α = 1.5406 Å.

All the electrochemical experiments were performed with an SP300 BioLogic potentiostat (Seyssinet-Pariset, France). The geometric surface of the samples was 0.75 cm^2^. The electrocatalytic activity was analyzed in a three-electrode cell with the Ni-Cu coating as a working electrode, a Pt foil as the counter electrode, and an SCE as the reference electrode (Figure 1c). The Linear Sweep Voltammetry (LSV) measurements ranged from the Open Circuit Potential (OCP) value to 1.5 V vs. the SCE in the non-stirred 1 M NaOH solution with a scan rate of 10 mV/s. The OCP was measured for the sample’s immersion time of 1 min. Based on the obtained curves, the Tafel slope and the onset potential (E_ONSET_) values were determined.

The contact angle measurements were performed using a high-speed camera Model: 9501 with HiBestViewer 1.0.5.1 software. A 10 μL droplet of deionized water was applied three times to the surface of each sample. The contact angle was determined through contour analysis utilizing the ImageJ software version 1.8.0.

## 3. Results and Discussion

### 3.1. Synthesis of Ni-Cu Alloys

**Figure 2:**
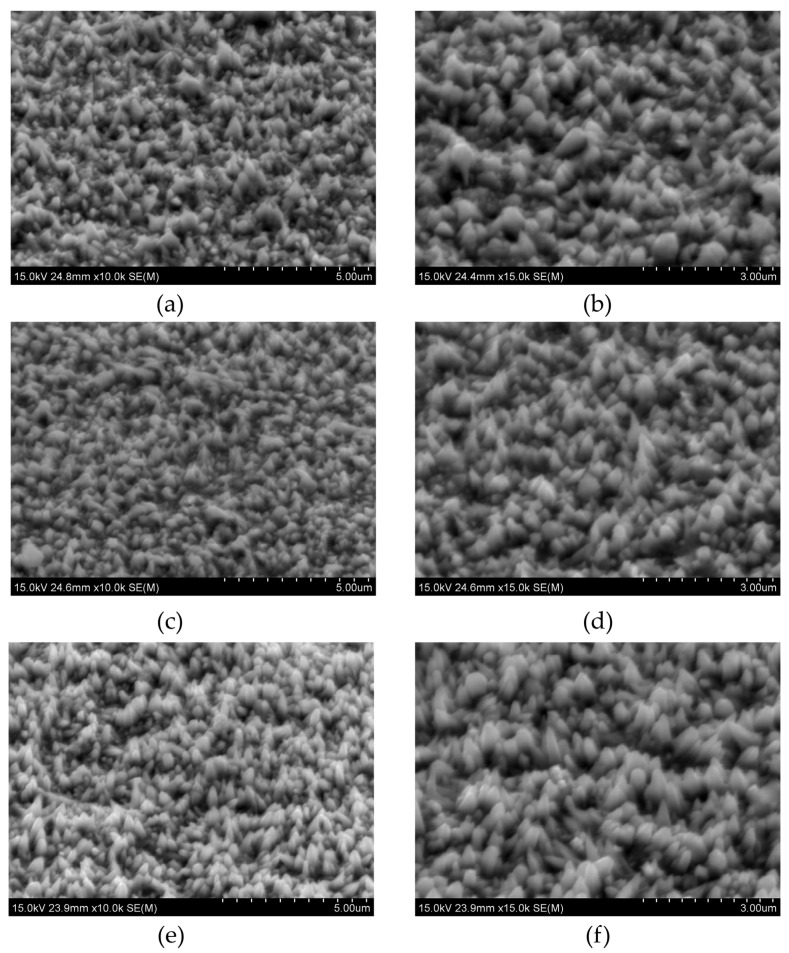
SEM photos of titled samples deposited from the electrolytes with the following CuCl_2_ concentration: (**a**,**b**) 0.01 mM, (**c**,**d**) 0.02 mM, and (**e**,**f**) 0.05 mM.

Firstly, different CuCl_2_ contents were added to NiCl_2_ solutions to synthesize Ni-Cu alloys. Copper chloride was chosen because it is believed that chloride ions act as a crystal modifier [43]. Table 1 lists the chemical composition of the obtained samples, and Figure 2 shows their morphology.

When the concentration of copper chloride is too low, Cu is not detected by EDS analysis. The detection limit of this technique is about 0.1 at.%. The morphology of the deposits remains conical, featuring smaller and larger cones. The detected chlorine is likely a residue from the electrolyte (Table 1). As the Cu concentration increases, the copper content reaches 2.1 at.% and 3.9 at.% for 0.02 mM and 0.05 mM CuCl_2_, respectively. The changes in the samples’ structure are also noticeable. For 0.02 mM CuCl_2_, the morphology is uniform, with most structures forming large cones. As the concentration increases, these large cones develop rounded ends (Figure 2). The Cl^−^ ions coming from NiCl_2_ are sufficient for synthesizing conical structures, but in many works, NH_4_Cl is added as a crystal modifier.

The electrolyte with a concentration of CuCl_2_ equal to 0.05 mM was chosen for further analysis of the crystal modifier’s influence on Ni-Cu alloys, as it allows the synthesis of a conical structure with sufficient Cu content.

**Figure 3:**
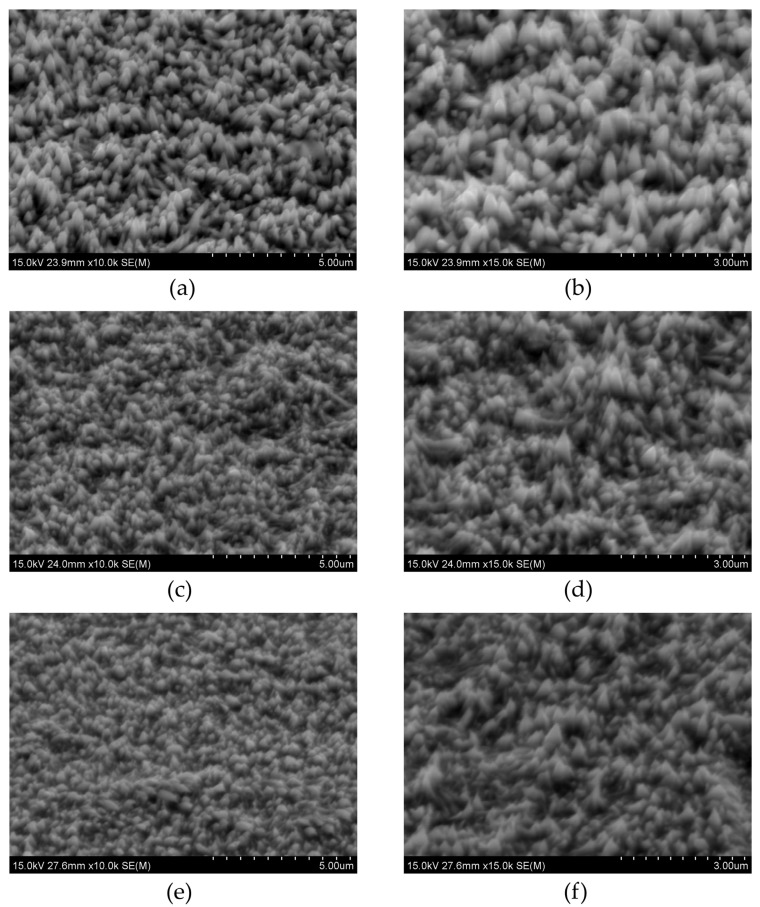
SEM photos of titled samples deposited from the electrolytes containing (**a**,**b**) 0 g/L, (**c**,**d**) 20 g/L, and (**e**,**f**) 40 g/L NH_4_Cl.

The crystal modifier NH_4_Cl in amounts of 20 g/L and 40 g/L was added to the electrolyte containing NiCl_2_ and CuCl_2_ (0.05 mM). The addition of the crystal modifier is usually listed in the literature in g/L; therefore, the same unit is kept in this work. The influence of its content on alloys’ chemical composition and morphology was analyzed using EDS (Table 2) and SEM (Figure 3) techniques.

The addition of the crystal modifier reduces the Cu content in the coatings. As more modifier is added, the loss of copper becomes more significant compared to samples deposited without NH_4_Cl. However, the morphology of the samples remains largely unaffected by the presence of this chemical component. When 20 g/L of ammonium chloride is introduced, cone growth is inhibited, resulting in smaller structures. Further addition of NH_4_Cl does not produce additional changes in the morphology. D. Goranova and others [44] stated that, due to the different deposition modes of Ni and Cu, nickel tends to deposit in the concave regions, while copper preferentially deposits on the convex parts.

**Figure 4:**
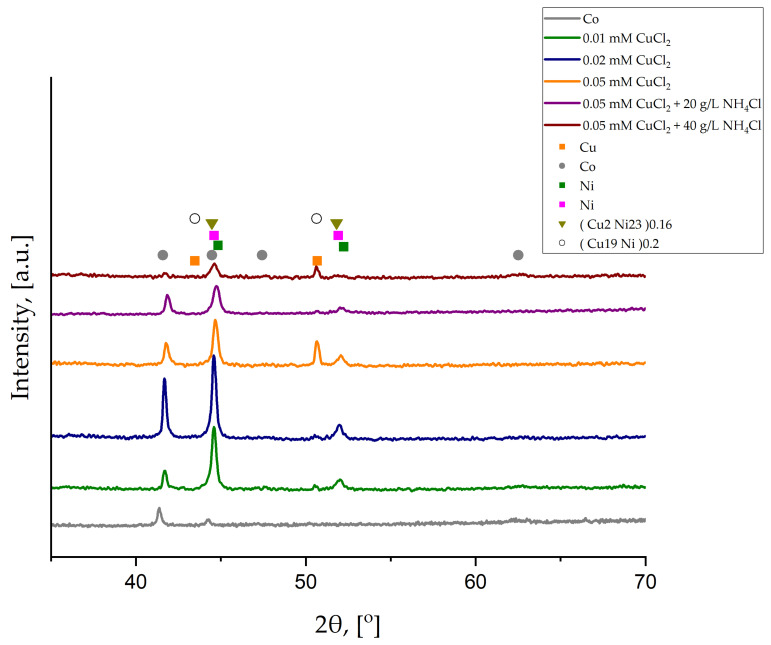
XRD diffraction patterns for all the synthesized samples.

To better characterize the influence of CuCl_2_ concentration and crystal modifier content on the deposition of Ni-Cu alloys, XRD analyses were performed. Results are shown in Figure 4.

The XRD diffraction pattern of the Cu/Co substrate shows that the thickness of the Co deposited layer was sufficient to block the Cu signal coming from the foil. All the deposited Ni-Cu coatings were too thin to cover the signal from the thick Co layer. However, peaks from Ni and Cu are present. Additionally, two different Cu-Ni phases are also detected. No transformation in the crystal structure is observed with increasing copper chloride concentration or crystal modifier content. However, the intensity of peaks at low 2θ angles (approximately 45° to 53°), attributed primarily to CuNi phases, decreases following the addition of NH_4_Cl, due to the reduced Cu content as shown in Table 2. All the structures, except for the Co layer, are cubic. The characteristics of each phase based on Joint Committee on Powder Diffraction Standards (JCPDS) cards are listed in Table 3.

The observed d-spacing of peaks that contributed to CuNi phases (approximately 45° to 53) are listed in Table 4 and compared with the data from JCPDS cards for (Cu2Ni23)0.16 and (Cu19Ni)0.2.

For most of the samples, phases Ni (Table 3) and (Cu2Ni23)0.16 are present, whereas for the sample deposited from the electrolyte containing 40 g/L NH_4_Cl, there is a mix of Ni and (Cu19Ni)0.2. Generally, the observed d-spacing is lower than the standard one, except for the sample deposited from the electrolyte with 40 g/L NH_4_Cl. For 20 g/L NH_4_Cl, the difference (0.0332) between the observed d-spacing and the standard value for (Cu2Ni23)0.16 (Table 4) is the largest. The observed d-spacing value can decrease due to the presence of compressive stress that can be, i.a., substrate-induced stress coming from the Co layer deposited on the Cu substrate (Figure 1a) or, for 20 g/L NH_4_Cl, the noticeable drop in Cu content, as listed in Table 2. When Cu-Ni alloys are brush-plated, Ni and Cu 3.8Ni phases are present [45]. At the same time, the co-deposition from the sulphate solution allows the fabrication of an alloy with the Cu50Ni50 phase [46].

**Figure 5:**
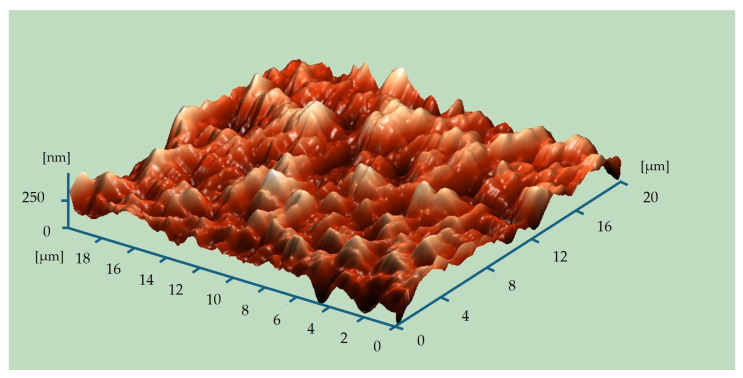
AFM 3D image of the sample deposited from the electrolyte containing 0.05 mM CuCl_2_.

**Figure 6:**
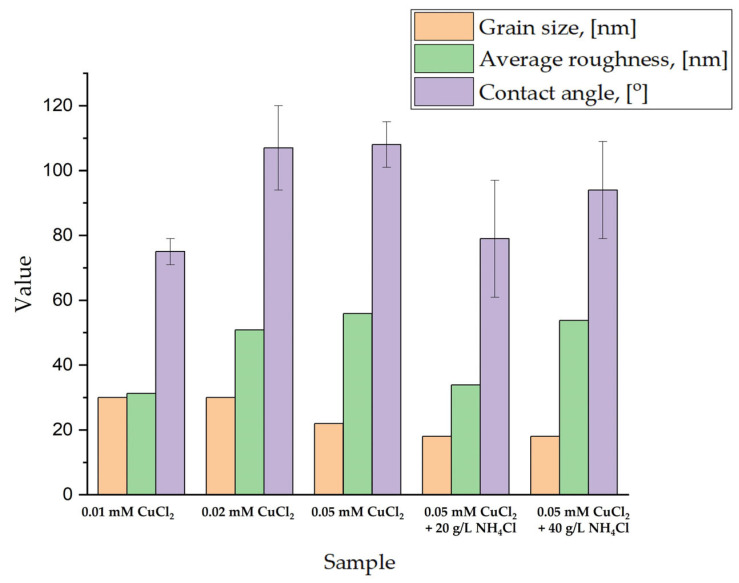
Summary of grain size, average roughness, and contact angle values.

Based on the obtained XRD diffraction patterns, the grain size was calculated using the Scherrer equation considering the peak at ~44.6^o^. This peak can be connected to Ni or CuNi phases. Additionally, the average roughness analysis was conducted using the AFM method. The scan area was 20 µm × 20 µm. An example of a 3D image is shown in Figure 5. Changes in the samples’ wettability as an important material’s property in the case of its application as a catalyst were also investigated. All these measured values are summarized in Figure 6.

With the increase in the copper chloride content, the grain size decreases. The addition of a crystal modifier causes a further decrease, irrespective of the amount added. A. Ijaz and others [47] deposited Ni coatings from a Watts bath in the presence of poly-(2-ethyl-2-oxazoline) (PEOX) for the grain refining effect. There was a clear decrease in nanometer grain sizes from ~1000 nm to ~12 nm. This phenomenon is associated with the control over the adatom mobility due to PEOX adsorption. In this work, however, the influence is relatively slight, as Cl^−^ ions from NiCl_2_, acting as a crystal growth modifier, had already affected the grain size. Instead, the average roughness S_a_ increased with the addition of CuCl_2_, which was confirmed by observations based on the SEM observations (Figure 2). With the addition of copper chloride, larger cones are observed. Subsequently, the addition of 20 g/L NH_4_Cl leads to a decrease in surface roughness, as the cones become smaller (Figure 2b,c). When the concentration of the capping agent is increased to 40 g/L, the roughness (S_a_) rises again to a value similar to that of the sample deposited without the capping agent. Except for the sample deposited from the electrolyte containing 0.01 mM CuCl_2_, which remains hydrophilic, all other samples exhibit wettability near the boundary between hydrophilic and hydrophobic behavior. In the case of pure Ni cones, the addition of 20 g/L of the crystal modifier increases the roughness S_a_ from ~67 to ~96 nm [48]. Its presence has an even greater effect on wettability, with the contact angle changing from about 81° to roughly 140°. Although Cl^−^ ions are already present in the electrolyte from NiCl_2_, the additional Cl^−^ from NH_4_Cl does not deteriorate the quality of the conical structures. Therefore, the growth mechanism presented in this work should vary from that of pure Ni cones. As mentioned, Cu could be deposited in the upper part of the cones and therefore influences the wettability of the samples.

**Figure 7:**
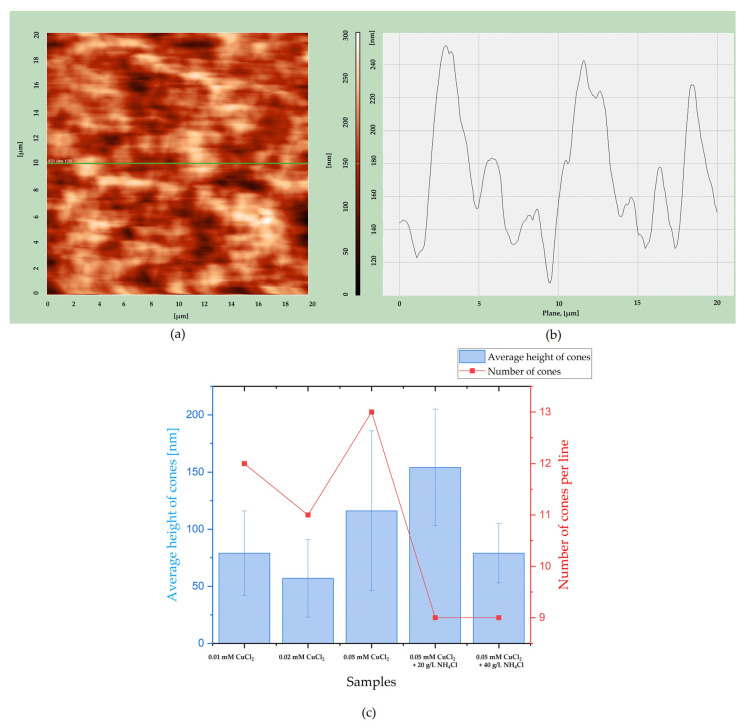
(**a**) Two-dimensional image from AFM with the 128 line marked green. (**b**) Cross-section line showing the height of cones. (**c**) Summarized average height of cones and number of cones for each sample.

Additionally, the average height of cones and the number were estimated using a cross-sectional line from a 2D AFM image, as shown in Figure 7. Using Nova AFM software (Nova 1.1.1 Revision 18376), line number 128 was chosen (Figure 7a), and the cross-section was obtained, as shown in Figure 7b. It allows the determination of average cone height and the number of cones per line number 128 for each sample and assessment of the geometric surface area of the alloys. The results are shown in Figure 7c.

There is no clear dependency between the average height of cones and the composition of electrolytes. The cones are of similar height. However, conical structures seem to be the most uniform for samples deposited from the solution containing 0.05 mM CuSO_4_ and 40 g/L NH_4_Cl, as the standard deviation is the smallest. The number of cones decreases when the crystal modifier is added, regardless of its content. The changes in cone numbers are slight. This result is consistent with the SEM photos shown in Figure 2 and Figure 3.

In the literature review [42], Cu cones were deposited from sulphate solutions using either pulsed electrodeposition or potentiostatic deposition, with NaCl and Janus Green B (JGB) added as crystal modifiers, respectively. The addition of JGB allows the synthesis of superhydrophobic structures [49]. In both cases, the cones show a strong (1 1 1) preferred orientation. However, the mechanism of conical growth remains not fully understood. Therefore, Y. Deng and others explain that this is based on screw dislocation theory [49], while M. Dong et al. proposed a twinning growth mechanism [50].

In this work, the conical growth of Ni-Cu alloys is interrupted when NH_4_Cl is added to the electrolyte. It is connected with the complexes created between Cu^2+^ and NH_3_ in the solution. Calculations supporting this explanation were performed using the IUPAC Stability Constants Database based on data published [51,52].

**Figure 8:**
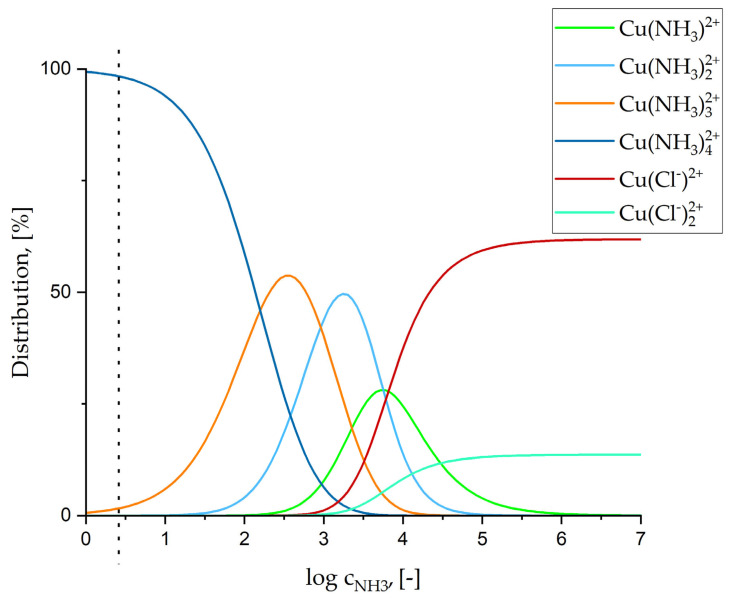
Function of copper (II) species’ distribution in aqueous solutions depending on the concentration of NH_4_Cl. Conditions: pH = 4; 0.05 mM Cu^2+^; 374 mM NH_3_; 2056 mM Cl^−^. The concentration of NH_3_ for 20 g/L NH_4_Cl is marked with the dotted line.

Figure 8 shows the distribution of copper (II) species in aqueous solutions as a function of the concentration of NH_4_Cl (c_NH3_). The full dissociation of NH_4_Cl was assumed.

As can be seen, nearly all Cu^2+^ ions form complexes with ammonia, which likely explains the decreased Cu content in the coating after the addition of NH_4_Cl. The ammonium chloride shows a weak acid solution nature as it is formed from the neutralization of a strong acid and a weak base. Its dissociation causes a slight lowering of the solution’s pH. Additionally, to deposit Ni-Cu alloys from alkaline citric electrolyte, a 25% NH_4_OH is often used in the adjustment of the electrolyte pH to 9 [44,53]. However, when the process is performed in sulphate solutions, the morphology is developed, but the conical structures are not present. This highlights the necessity of chlorine ions to synthesize cones.

### 3.2. Electrocatalytic Properties of the Coatings

**Figure 9:**
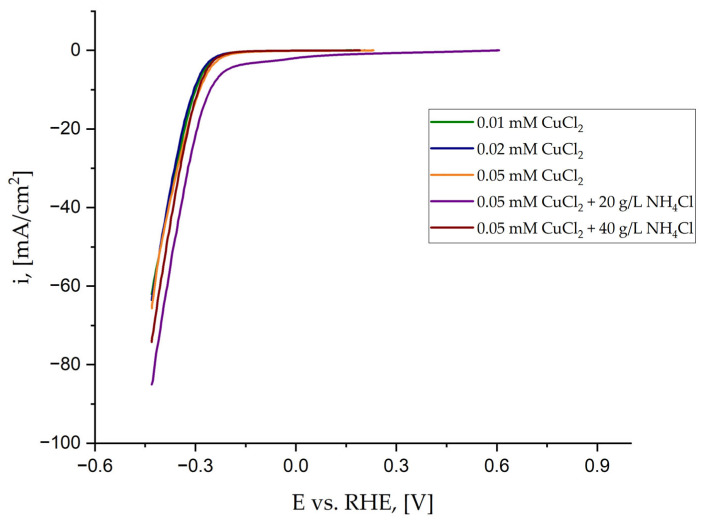
LSV curves obtained in 1 M NaOH with 85% iR compensation.

The detailed characterization of coatings’ electrocatalytic properties was carried out in 1 M NaOH by Linear Sweep Voltammetry. Measurements were performed after the application of 85% iR compensation. This compensation allows for more accurate evaluation by correcting for the voltage loss caused by the electrolyte solution. Because SCE was used as the reference electrode, the potential recorded against the saturated calomel electrode was converted to the reversible hydrogen electrode (RHE). The obtained curves are shown in Figure 9.

All coatings show similar properties; however, the catalytic activity is slightly enhanced for the sample deposited by the electrolyte containing 0.05 mM CuCl_2_ and 20 g/L NH_4_Cl. To make sure that the electrode was stable, the LSV measurements were repeated three times consecutively. An example of the obtained curves for the alloy deposited from the solution containing 0.05 mM CuCl_2_ and 40 g/L NH_4_Cl is shown in Figure S1. A slight decrease in the catalytic properties can be observed, but the electrode stability is satisfactory.

**Figure 10:**
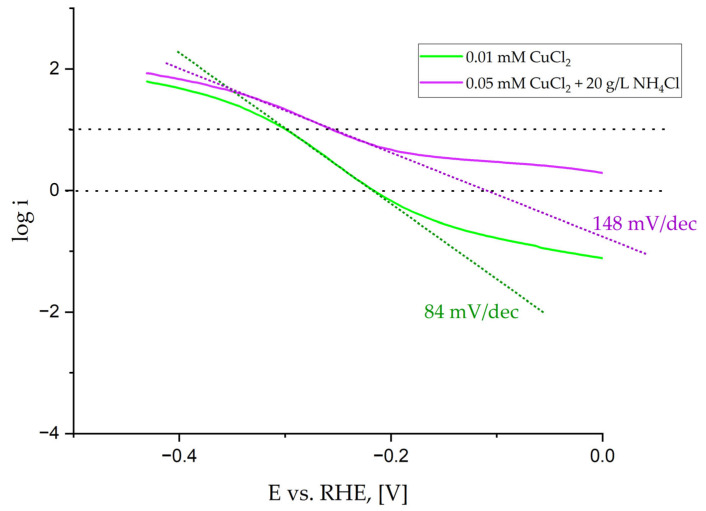
Determination of Tafel slope.

Therefore, to better characterize coatings, typical parameters such as the values of the current density at fixed overpotentials of −200 and −400 mV vs. RHE, the potential required to reach i = 10 mA/cm^2^, Tafel slopes, and E_ONSET_ potential were determined based on curves shown in Figure 6 and listed in Table 5. The error is about ±1%. To determine the potential E_ONSET_, at which the hydrogen evolution reaction begins, two tangents were plotted on the curve of the potential dependence on the current density. The Tafel slope was determined in the range of low current density from 1 to 10 mA/cm^2^. An example of the curves is shown in Figure 10.

We performed a detailed analysis that shows similar catalytic properties of all deposited coatings. The difference appears in the values of the Tafel slope, where samples deposited by the electrolyte containing 20 g/L NH_4_Cl show a different mechanism. For the Tafel slope higher than 120 mV/dec [54], the reaction follows the Volmer (RDS)–Tafel pathway when the diffusion rate is much faster than the hydrogen oxidation reaction (HOR) rate. For Tafel slopes in the range of 60 and 120 mV/dec, a more prominent HOR current and a lower Tafel slope near the equilibrium potential appear due to slower H_2_ mass transport and an increase in the concentration of the locally trapped H_2_ in the catalyst layer.

The hydrogen evolution started the earliest on the sample deposited by the solution containing 20 g/L NH_4_Cl, as seen in Figure 10. However, the determined values are similar for all samples. This could be expected as the wettability of the coatings is also similar, even though the sample deposited by the electrolyte containing 0.01 mM CuCl_2_ is the only one that is hydrophilic. Moreover, the height of the cones is similar, and their number does not change much. Due to the slight differences between Cu content from 1.6 to 3.9 at.% (Table 2), the catalytic properties vary just slightly. Therefore, the described fabrication method of Ni alloy cones using the most common crystal modifier NH_4_Cl is limited to the metals, which does not create complexes with NH_4_Cl at pH = 4, like Co [55]. Cobalt creates complexes with ammonia, but at higher pH (>9) [56]. For that reason, the proposed method of Ni-Cu conical structures synthesis from the electrolyte containing the addition of NH_4_Cl seems to be adverse. The present crystal modifier creates complexes with Cu^2+^ ions. The cones’ fabrication performed from the electrolyte containing only nickel and copper chloride salts is sufficient. Compared with the Ni-Cu cones obtained by the annealing of the Ni cones covered with a thin Cu layer [27], the Tafel slopes obtained in this work are significantly lower, besides the sample synthesized from the electrolyte containing 0.05 mM CuCl_2_ and 20 g/L NH_4_Cl, which is comparable with the samples before annealing and follows the Volmer (RDS)–Tafel pathway. This means that the co-deposition of Ni-Cu from the electrolyte, rather than later deposition of Cu on synthesized Ni cones, leads to the fabrication of a material change in the mechanism of hydrogen evolution. Hydrogen evolution started slightly earlier, when Cu was deposited up to 10 sec on previously prepared conical structures.

The obtained data were compared with the already published results on the Ni and Ni alloy Tafel slopes listed in Table 6. The values chosen are the lowest Tafel values declared in each article.

Compared with the values of the Tafel slope of other coatings presented in the literature, the Ni-Cu cones synthesized in this work show satisfactory properties, placing them in the middle of the range of values. However, considering the simplicity of their synthesis, the results are promising.

## 4. Conclusions

Performed experiments allow the synthesis of Ni-Cu cones characterized by promising catalytic activity, considering the low-cost and fast one-step method. However, the addition of NH_4_Cl, leading to the formation of $\mathrm{Cu}{\left({\mathrm{NH}}_{3}\right)}_{3}^{2+}$ and $\mathrm{Cu}{\left({\mathrm{NH}}_{3}\right)}_{4}^{2+}$ complexes, suppressed Cu deposition. The number of cones was slightly lower as well. Therefore, the synthesis of conical structures through the simple addition of CuCl_2_ into the electrolyte containing NiCl_2_ and H_3_BO_3_ proves to be an effective approach, even though the resulting differences in catalytic properties are slight. The influence of Cl^−^ ions coming from the nickel chloride is so strong that it allows the synthesis of Ni-Cu alloys containing ~4 at.% Cu without the addition of an additional crystal modifier. Further research on the one-step method is required to highlight the limitations of this method. It is especially important due to the simplicity and low-cost character of this approach, as it can be successfully used in the improvement of materials’ catalytic properties [68].

## Figures and Tables

**Table 1:** EDS results for the deposited alloys.

Concentration of CuCl_2_ [mM]	Chemical Composition [at.%]			
	Ni	Cu	O	Cl
0.01	92.2 ± 4.6	0.0	4.6 ± 0.2	3.2 ± 0.2
0.02	95.1 ± 4.8	2.1 ± 0.1	2.8 ± 0.1	-
0.05	90.8 ± 4.4	3.9 ± 0.2	5.3 ± 0.3	-

**Table 2:** Chemical composition of the deposited alloys.

Content of Crystal Modifier [g/L]	Chemical Composition [at.%]			
	Ni	Cu	O	Cl
0	90.8 ± 4.5	3.9 ± 0.2	5.3 ± 0.3	-
20	91.2 ± 4.6	1.6 ± 0.1	7.2 ± 0.4	-
40	95.5 ± 4.8	1.7 ± 0.9	2.7 ± 0.1	-

**Table 3:** Parameters for each phase mentioned in Figure 3.

Formula	a [Å]	2θ [^o^]	h k l	d-Spacing [Å]	Card Number
Cu	3.6077	43.47	1 1 1	2.0800	00-003-1018
		50.67	2 0 0	1.8000	
Co	2.5060	41.58	1 0 0	2.1700	01-089-7373
		44.47	0 0 2	2.0360	
		47.43	1 0 1	1.9150	
		62.51	1 0 2	1.4850	
Ni (green points)	3.5140	44.83	1 1 1	2.0200	00-003-1051
		52.23	2 0 0	1.7500	
Ni	3.5175	44.60	1 1 1	2.0300	00-001-1260
		51.91	2 0 0	1.7600	
(Cu2Ni23)0.16	3.5260	44.47	1 1 1	2.0360	01-077-7710
		51.81	2 0 0	1.7630	
(Cu19Ni)0.2	3.6025	43.47	1 1 1	2.0800	01-077-7712
		50.64	2 0 0	1.8010	

**Table 4:** Comparison of observed and standard d-spacing.

Concentration of CuCl_2_ [mM]	Content of Crystal Modifier [g/L]	2θ [^o^]	Observed d-Spacing [Å]	Phase	2θ [^o^]	Standard d-Spacing [Å]	Card Number
0.01	0	44.56	2.0319	(Cu2Ni23)0.16	44.47	2.0360	01-077-7710
		52.01	1.7570				
0.02	0	44.56	2.0315				
		51.90	1.7605		51.81	1.7630	
0.05	0	44.64	2.0284				
		52.09	1.7545				
	20	45.24	2.0028	(Cu19Ni)0.2	43.47	2.0800	01-077-7712
		52.55	1.7400				
	40	44.63	2.0287		50.64	1.8010	
		50.61	1.8021				

**Table 5:** Electrochemical parameters for Ni-Cu alloys measured with 85% iR compensation, with an iR drop of approximately 3.4 Ω.

Concentration of CuCl_2_ [mM]	Content of Crystal Modifier [g/L]	j at η = −200 mV [mA/cm^2^]	j at η = −400 mV [mA/cm^2^]	E at i = −10 mA/cm^2^ [V vs. RHE]	Tafel Slope [mV/dec]	E_ONSET_ [V vs. RHE]
0.01	0	−0.65	−49.2	−0.29	84	−0.286
0.02	0	−0.65	−51.1	−0.30	89	−0.295
0.05	0	−1.39	−50.4	−0.29	85	−0.284
	20	−5.13	−70.1	−0.26	148	−0.271
	40	−0.65	−58.4	−0.29	79	−0.282

**Table 6:** Literature review on Tafel slopes of Ni and its alloys.

Material	Medium	Tafel Slope [mV/dec]	REF
Ni-Cu	1 M NaOH	79	This work
Ni-Cu	1 M NaOH	143	[57]
Ni-Cu	1 M KOH	149	[58]
Ni-Cu cones	1 M NaOH	107	[27]
Pure Ni net	30 wt.% aqueous KOH solution	127	[59]
Raney-Ni		122	
Amorphous Ni-C	1 M NaOH	146	[60]
Ni-Cu alloy nanosheets	1 M KOH	57	[61]
3D Cu/Ni nanostructures	1 M NaOH	121	[62]
Ni-Cu	1 M KOH	120	[63]
Ni(Cu)/NF	1 M KOH	33	[64]
Ni-Cu dendrides on NF	1 M KOH	82	[65]
Ni 1.8Cu0.2-P nanosheets on NF	1 M KOH	70	[66]
Cu–Ni/Ni–Cu	1 M KOH	79	[67]

NF—Nickel Foam.

## Data Availability

The original contributions presented in this study are included in the article/Supplementary Material. Further inquiries can be directed to the corresponding author.

## Supplementary Materials

The following supporting information can be downloaded at: https://www.mdpi.com/article/10.3390/ma18112499/s1, Figure S1: Curves obtained after 3 consecutive measurements for sample deposited from the solution containing 0.05 mM CuCl_2_ and 40 g/L NH_4_Cl.

## Abbreviations

The following abbreviations are used in this manuscript:

HER	Hydrogen Evolution Reaction
SEM	Scanning Electron Microscope
EDS	Energy Dispersive X-ray Spectrometer
XRD	X-ray Diffraction
AFM	Atomic Force Microscope
SCE	Saturated Calomel Electrode
OCP	Open Circuit Potential
E_ONSET_	Onset Potential
PEOX	poly-(2-ethyl-2-oxazoline)
JGB	Janus Green B
RHE	Reversible Hydrogen Electrode
HOR	Hydrogen Oxidation Reaction
JCPDS	Joint Committee on Powder Diffraction Standards
