# Supplementary material for: Influence of Crystal Modifier Content on Ni-Cu Catalysts Dedicated to the Hydrogen Evolution Reaction
Source: Materials (Basel). 2025 May 26;18(11):2499. doi: 10.3390/ma18112499 (PMC12155775; doi:10.3390/ma18112499)
Supplement: Supplementary file 1 [file materials-18-02499-s001.zip › materials-3646798-supplementary.pdf]

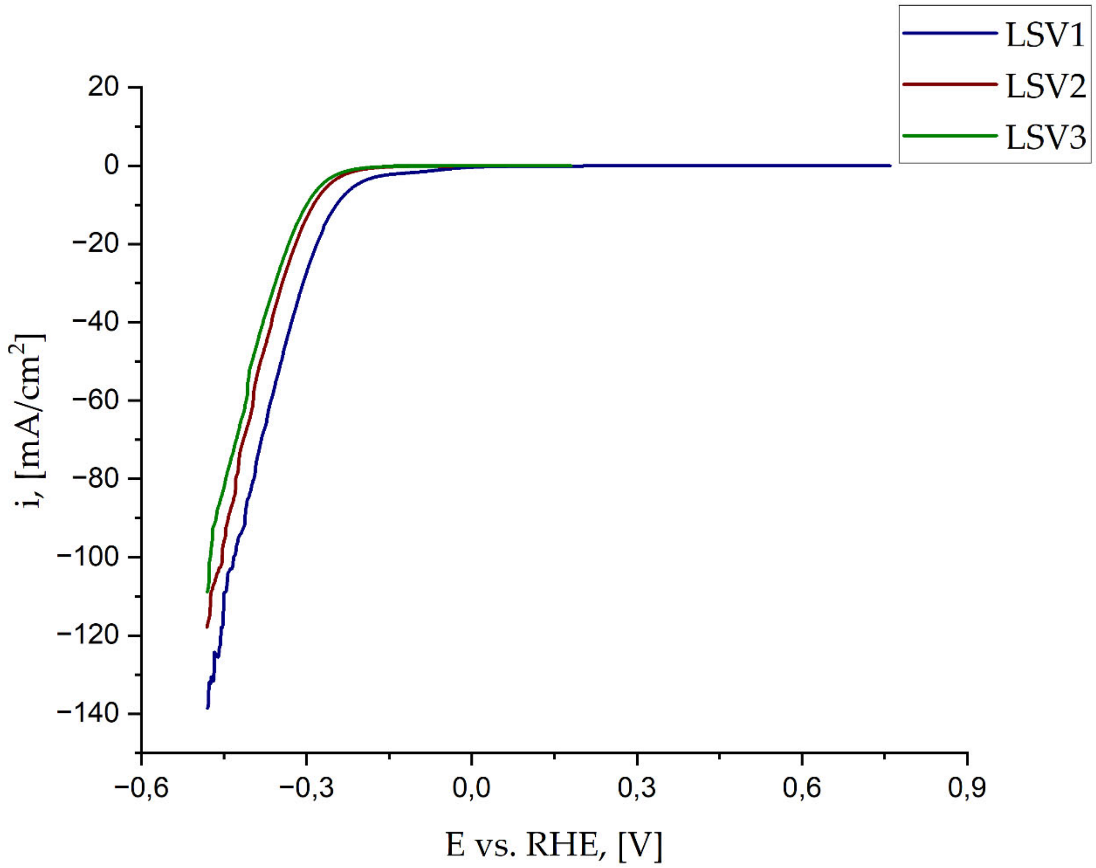


**Figure S1.** Curves obtained after 3 consecutive measurements for sample deposited from the solution containing 0.05 mM $CuCl_2$ and 40 g/L $NH_4Cl$.
